# Psychometric evaluation of the French version of the questionnaire attitudes towards morphine use; a cross-sectional study in Valais, Switzerland

**DOI:** 10.1186/1472-6955-13-1

**Published:** 2014-01-10

**Authors:** Maria Ferreira, Henk Verloo, Cédric Mabire, Margarida Maria S Vieira, Pedro Marques-Vidal

**Affiliations:** 1 Hôpital de Sion, Avenue Grand-Champsec 80, Case Postale 736, Sion, 1951, Switzerland; 2 Haute Ecole de Santé La Source, Avenue Vinet 30, Lausanne, 1004, Switzerland; 3 Haute Ecole de Santé Vaud, Unité de recherche en Santé, Av. de Beaumont 21, Lausanne, 1011, Switzerland; 4 Universidade Católica Portuguesa, Instituto de Ciências da Saúde, Palma de Cima, Lisboa, 1649-023, Portugal; 5 Institute of Social and Preventive Medicine, University Hospital of Lausanne, route de la Corniche 10, Lausanne, 1010, Switzerland

**Keywords:** Instrument validation, Morphine use, Attitudes, Psychometrics, Switzerland

## Abstract

**Background:**

In Switzerland, nurses are allowed to prescribe and administer morphine in emergency situations without a doctor. Still, nurses and other health professionals are often reluctant to prescribe and administer morphine for pain management in patients. No valid French-speaking instrument is available in Switzerland to assess the attitudes of nurses and other health professionals towards the prescription and administration of morphine. In this study, we evaluated the psychometric properties of the French version of the questionnaire “Attitudes towards morphine use”.

**Methods:**

The instrument was derived from an Italian version. Forward and back translations of the questionnaire were performed. Item analysis and construct validity were assessed between April and December 2010 in a cross sectional study including five Swiss hospitals in a sample of 588 health professionals (533 nurses, mean age 38.3 ± 10.2 years). Thirty subjects participated in test-retest reliability.

**Results:**

The time to complete the instrument ranged between 12 and 15 minutes and neither floor nor ceiling effect were found. The initial 24-item instrument showed an intraclass correlation (ICC) of 0.69 (95% CI: 0.64 to 0.73, P < 0.001), and a Cronbach’s α of 0.700. Factor analysis led to a six-component solution explaining 52.4% of the total variance. After excluding five items, the shortened version showed an ICC of 0.74 (95% CI, 0.70 to 0.77, P < 0.001) and a Cronbach’s α of 0.741. Factor analysis led to a five-component solution explaining 54.3% of the total variance. The five components were named “risk of addiction/dependence”; “operational reasons for not using morphine”; “risk of escalation”; “other (non-dependence) risks” and “external (non-operational) reasons”. In test-retest, the shortened instrument showed an ICC of 0.797 (95% CI, 0.630 to 0.911, P < 0.001) and a Cronbach’s α of 0.797.

**Conclusions:**

The 19-item shortened instrument assessing attitudes towards the prescription and administration of morphine showed adequate content and construct validity.

## Background

In hospitals, four out of five patients present with acute or chronic pain [1]. Pain management supposes far more than the simple prescription and administration of analgesic drugs, namely morphine and its derivatives [2,3]. Indeed, the health professionals’ behavior is influenced by opposite factors such as the intention to completely relieve pain [2,4] and many nonmedical factors such as concerns regarding the deleterious health effects of morphine administration and the (unfounded) fear of legal consequences from possible deleterious effects [5-7].

Although several evidence-based guidelines have been issued [2,8,9], inadequate attitudes towards morphine administration for pain relief (opiophobia) are still observed among health professionals [4,10-12]. Opiophobia can be defined as a set of inappropriate attitudes and beliefs regarding the deleterious effects of morphine administration for pain relief such as death, addiction, respiratory depression or urinary retention [13-16]. The main reasons for these inappropriate attitudes and beliefs are the lack of knowledge regarding morphine administration, a negative opinion about morphine due to substance abuse and the risk of developing addiction during morphine administration [11,17]. Hence, it is necessary to adequately characterize the beliefs and attitudes of health professionals regarding the prescription and administration of morphine and its derivatives. Several instruments have been proposed to assess attitudes regarding morphine prescription for pain relief [5-7,18] but after a thorough literature search none has been adapted to French. Further, there is little information regarding their psychometric evaluation, even for non-French instruments [5,6].

The “Attitudes face à l’utilisation de la morphine (AUM)” [Attitudes towards the use of morphine] was initially developed in 2003 by Musi & Bionaz to assess attitudes towards the use and prescription of opioids as analgesic by nurses and doctors in the Italian-speaking Swiss canton of Tessin [7]. Most of these attitudes and beliefs have also been reported in other studies [19-22]. A Portuguese version has been applied among Portuguese health professionals of the Beira Interior region, South-East Portugal [12,23] and a French version was recently developed and applied among student nurses in French-speaking Switzerland [24]. This instrument was preferred because it was available in three different languages (Italian, Portuguese and French), which would theoretically allow comparisons between countries. Further, literature search provided no other instrument assessing morphinophobia for Portuguese or French-speaking countries. Still, no thorough psychometric evaluation was performed.

Thus, the aim of this study was to analyze the psychometric properties of the French version of the “Attitudes face à l’utilisation de la morphine (AUM)” [Attitudes towards the use of morphine].

## Methods

### Data collection and procedure

The study was approved by the Internal Board Committee of the Wallis Hospital Center (Hôpital du Valais). The listing of all nurses and doctors was obtained from the human resources of the five hospitals, then a random sample of 1100 persons was drawn and the questionnaires were sent to them. Briefly, the questionnaires were sent to the different departments, and distributed to the persons on duty during a single day of the week. The day of sampling was decided by each head of the department. All participants gave their written informed consent before completing the instrument. The instrument was a self-administered tool and all completed instruments were anonymized prior to analysis and the completed instruments were kept in a locked room with restricted access.

Data were collected between April and December 2010 using a cross-sectional design conducted in five hospitals of the Swiss Canton of Valais: Sierre, Sion and Martigny hospitals, Clinique de St Claire and Centre Valaisan Pulmonaire. The heads of the departments were contacted and informed about the aim and the methodology of the study.

### Instrument

The development of the original instrument (in Italian) has been described previously [7]. Briefly, it is composed of 26 items formulated as statements about prescription and administration of morphine, and the answers are provided in a 5-point Likert scale. All statements are scored in the same direction, i.e. ranging from 1 = “Totally disagree” to 5=“Totally agree”. A global score is derived by summing up all the responses, with a theoretical distribution of 104 possible score values, ranging between 26 and 130. The French version of the instrument was initially forward translated from Italian and then back-translated to ensure reliability of the statements. A first analysis [24] showed that two items, “It is necessary to evaluate pain (using a visual scale)” and ”The doctor must inform the patient when prescribing a drug/medicine containing morphine”, provided no information; hence, a 24-item instrument was developed and the current study is based on the 24-item French version. Also in this first analysis, of the questionnaire, the measured time to complete ranged between 20 and 25 minutes.

### Data analysis

Statistical analysis was conducted using SPSS version 20.0 (IBM-SPSS, Armonk, NY, USA). Practicality was assessed by the time needed to complete the instrument and by feasibility, defined as the number of missing items [25]. The percentages of participants who scored at the floor (the worst 10% of score for the scale) or at the ceiling (the highest 10% of score for the scale) were also examined [26].

Reliability was assessed by the intra-class correlation coefficient (ICC) and its 95% confidence interval. We defined reliability as an extent to which a variable or set of variables is consistent in what it is intended to measure [27]. The internal consistency of statements was evaluated using Cronbach’s α coefficient. Internal consistency is defined as the correlation between the different statements of the instrument [27]. Cronbach values in the range of 0.81–1.00 indicate 'almost perfect’ agreement with 0.61–0.80 indicating 'substantial’, 0.41–0.60 'moderate’, 0.21–0.40 'fair’, 0.00–0.20 'slight’ and ≤0.00 indicates 'poor’ agreement [27,28]. Intra-class correlation coefficients can be interpreted in the same manner. Reliability assessment was conducted in two stages: 1) using the 24-item instrument and 2) using the shortened version after item elimination. A similar analysis of the shortened version was performed after stratifying on profession (nurses and doctors).

Validity was assessed by factor analysis. We defined validity as an extent to which a measure or set of measures correctly represents the concepts of the study [27]. An exploratory factor analysis of the 24-item instrument was conducted. Bartlett’s test of sphericity was used to assess if factor analysis was appropriate with the data analyzed. The Kaiser-Meyer-Olkin (KMO) index was used as a measure of sample adequacy, with KMO values ≥0.80 for conducting exploratory factor analysis. [27,29]. The following criteria were employed to determine the optimal number of factors to extract: 1) scree plot observation; 2) Kaiser’s *eigenvalue* >1 and 3) total variance explained >50% [27]. The following statistical criterion was used to identify items eligible for elimination: low communality (<0.35) and total variance explained <50% [27]. Factor analysis with varimax rotation and Kaiser normalization was used as this method assumes that the explained variances among factors do not overlap [30]. After item elimination, another factor analysis was conducted to assess the validity of the shortened version.

Stability was evaluated using the test–retest procedure. As recommended by Hair et al. [27], one month after the first test a convenience sample of thirty subjects (26 nurses, 4 doctors) were enrolled for the test-retest reliability within the two departments of the same hospital (Sierre) and the time needed to complete the questionnaire was measured by one of the investigators (MF). Reliability was assessed using Pearson correlation coefficient.

## Results

One thousand one hundred questionnaires were simultaneously distributed to the nurses and the doctors of the five hospitals, of which 588 (response rate: 53.5%, 533 nurses and 55 doctors) were returned. The mean age of the 588 participants was 38.3 years (SD = 10.2, range: 20–63). Most were women (84.0%) and nurses (90.6%), and the number of years as healthcare professionals (mean ± standard deviation) was 13.9 ± 10.0 (median 12, interquartile range 5–20).

**Figure 1:**
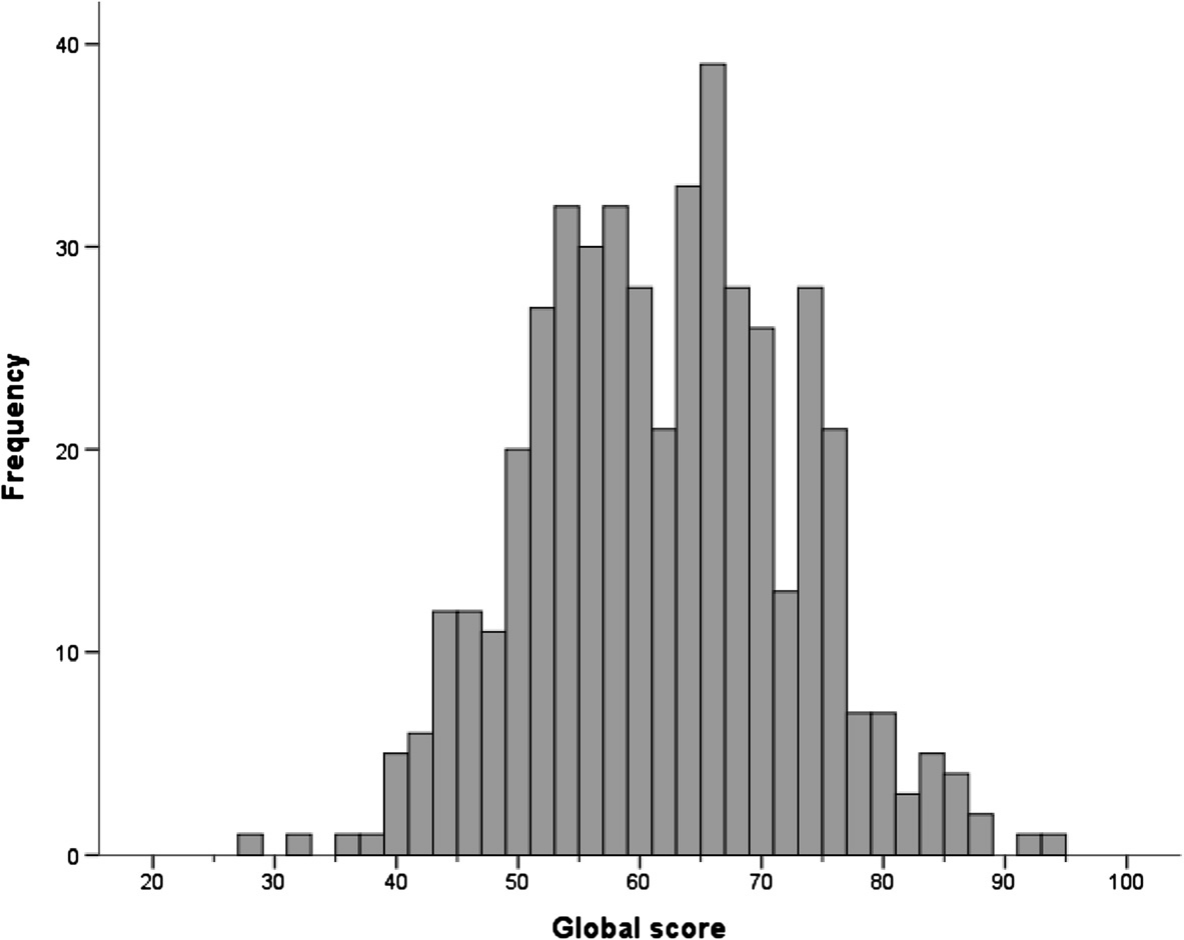
Distribution of the overall score for the 24-item instrument “Attitudes towards morphine use”, French version.

Overall, 139 participants (23.6%) had at least one missing answer which precluded the calculation of the overall score. Examination of the floor and ceiling effects indicated neither floor nor ceiling effect in the overall score (Figure 1).

### Original instrument (24-item version)

Regarding reliability, the 24-item instrument showed an ICC of 0.69 (95% CI: 0.64 to 0.73, P < 0.001).

The results of internal consistency for the whole instrument and the two constructs are summarized in Table 1. The 24-item instrument showed a Cronbach’s α of 0.700 and three items (5, 12 and 14) showed an improvement of internal consistency if removed; they are indicated in bold in Table 1. The English translation of the statements has not been psychometrically validated; please refer to the Additional file 1 for the valid French terms.

**Table 1:** 24-item French version of the Attitudes towards morphine use questionnaire: internal consistency (n = 458)

		Average*	**SD**	**Item-total correlation**	**Alpha if item deleted**
1	It means it is serious	2.03	1.20	0.309	0.670
2	It decreases life expectancy	1.68	1.07	0.240	0.676
3	[The patient] can get used quickly and one takes the risk of increasing the dose	2.39	1.27	0.512	0.651
4	Once treatment is initiated, there is the risk of being unable to stop	1.67	1.05	0.439	0.661
5	All patients can be prescribed morphine regardless of the type of pain	2.71	1.49	-0.013	0.701
6	The early use of morphine makes it difficult to use any other treatment in severe pain	2.48	1.39	0.397	0.660
7	IV administration is more effective than oral administration	3.40	1.45	0.199	0.680
8	The patients are against the prescription of morphine	2.63	1.13	0.248	0.675
9	The prescription of morphine means that there is no life expectation	1.36	0.94	0.215	0.678
10	There are other more effective drugs, hence the use of morphine is not justified	2.19	1.25	0.203	0.679
11	It is difficult to use and dose morphine	1.93	1.16	0.295	0.671
12	For some types of pain, it is necessary to use morphine	3.91	1.56	-0.053	0.706
13	Morphine is a drug of last resort	1.90	1.23	0.397	0.662
14	One can stop taking morphine whenever one wants to	2.90	1.42	-0.055	0.704
15	The prescription of morphine should be avoided for terminally ill patients	1.22	0.75	0.188	0.680
16	Sensation of pain decreases with age in the elderly, which does not justify its use	1.50	1.08	0.085	0.687
17	Risk of drug addiction	2.71	1.46	0.398	0.659
18	Risk of delirium or euphoria	3.15	1.38	0.311	0.669
19	Risk of drowsiness and sedation	3.91	1.19	0.252	0.675
20	Risk of respiratory depression	3.87	1.22	0.309	0.670
21	Legal risk compared to other drugs	3.02	1.52	0.316	0.668
22	Risk of physical and/or psychological dependence	3.09	1.35	0.400	0.660
23	Risk of discrimination	2.36	1.48	0.137	0.686
24	Risk of urinary retention	3.30	1.39	0.197	0.680

^*^possible values from 1 to 5.

The results for validity showed a statistically significant Bartlett’s test of sphericity (2551.47, P <0.001) and an adequate KMO value (0.81). The initial explorative analysis on the 24-item instrument resulted in a six-factor rotated solution explaining 52.4% of the total variance (not shown). Items 10 and 15 showed an extraction communality <0.35 and were subsequently removed.

### Shortened instrument (19-item version)

After removing 5 items, the psychometric properties of the shortened 19-item version of the instrument were assessed. Of the 588 participants, 117 (19.9%) had at least one missing answer precluding the calculation of the overall score.

Regarding reliability, the shortened instrument showed an ICC of 0.74 (95% CI, 0.70 to 0.77, P < 0.001). After sample stratification according to profession, the values were 0.74 (95% CI: 0.70 to 0.78, p < 0.001) for nurses and 0.74 (95% CI: 0.60 to 0.84, p < 0.001) for doctors.

The results of internal consistency for the shortened instrument are summarized in Table 2**.** The shortened instrument showed a Cronbach's α of 0.741 and no item improved Cronbach’s α upon removal. After sample stratification according to profession, the values for Cronbach’s α was 0.739 for nurses and 0.760 for doctors (see also Additional file 2).

**Table 2:** Shortened 19-item French version of the Attitudes towards morphine use questionnaire: internal consistency (n = 485)

		Average*	**SD**	**Item-total correlation**	**Alpha if item deleted**
1	It means it is serious	2.02	1.19	0.312	0.727
2	It decreases life expectancy	1.68	1.07	0.249	0.731
3	[The patient] can get used quickly and one takes the risk of increasing the dose	2.37	1.27	0.511	0.709
4	Once treatment is initiated, there is the risk of being unable to stop	1.66	1.04	0.445	0.718
6	The early use of morphine makes it difficult to use any other treatment in severe pain	2.48	1.39	0.413	0.717
7	IV administration is more effective than oral administration	3.37	1.45	0.211	0.736
8	The patients are against the prescription of morphine	2.64	1.13	0.198	0.735
9	The prescription of morphine means that there is no life expectation	1.35	0.92	0.206	0.734
11	It is difficult to use and dose morphine	1.93	1.16	0.326	0.726
13	Morphine is a drug of last resort	1.91	1.23	0.400	0.719
16	Sensation of pain decreases with age in the elderly, which does not justify its use	1.49	1.07	0.121	0.740
17	Risk of drug addiction	2.70	1.45	0.385	0.720
18	Risk of delirium or euphoria	3.15	1.37	0.295	0.728
19	Risk of drowsiness and sedation	3.91	1.18	0.262	0.731
20	Risk of respiratory depression	3.86	1.23	0.327	0.725
21	Legal risk compared to other drugs	3.02	1.52	0.365	0.722
22	Risk of physical and/or psychological dependence	3.09	1.35	0.407	0.718
23	Risk of discrimination	2.35	1.47	0.201	0.738
24	Risk of urinary retention	3.29	1.40	0.216	0.735

Item numbering corresponds to the original 24-item instrument.

^*^possible values from 1 to 5.

The results for validity are summarized in Table 3. The English translation of the statements has not been psychometrically validated; please refer to the Additional file 1 for the valid French terms. The shortened version of the instrument showed a significant Bartlett’s test of sphericity (1982.0, P <0.001) and a KMO value of 0.79. A five-factor rotated solution was obtained, explaining 54.3% of the total variance. The five constructs were named “risk of addiction/dependence”; “operational reasons for not using morphine”; “risk of escalation”; “other (non-dependence) risks” and “external (non-operational) reasons”. In nurses, the shortened version of the instrument showed a significant Bartlett’s test of sphericity (1791.8, p < 0.001), a KMO value of 0.78 and a five-factor rotated solution explaining 54.4% of the total variance. In doctors, the shortened version of the instrument showed a significant Bartlett’s test of sphericity (316.9, p < 0.001), a KMO value of 0.60, and a five-factor rotated solution explaining 64.9% of the total variance.

**Table 3:** Results of the factor analysis of the shortened 19-item instrument

		**1**	**2**	**3**	**4**	**5**
	**“Risk of addiction/dependence” (eigenvalue = 3.580, % variance explained = 18.8)**					
17	Risk of drug addiction	0.776				
18	Risk of delirium or euphoria	0.700				
19	Risk of drowsiness and sedation	0.543				
22	Risk of physical and/or psychological dependence	0.770				
	**“Operational reasons for not using morphine” (eigenvalue = 2.905, % variance explained = 15.3)**					
9	The prescription of morphine means that there is no life expectation		0.522			
11	It is difficult to use and dose morphine		0.662			
13	Morphine is a drug of last resort		0.516			
21	Legal risk compared to other drugs		0.698			
23	Risk of discrimination		0.707			
	**“Risk of escalation” (eigenvalue = 1.656, % variance explained = 8.7)**					
1	It means it is serious			0.598		
2	It decreases life expectancy			0.698		
3	[The patient] can get used quickly and one takes the risk of increasing the dose			0.654		
4	Once treatment is initiated, there is the risk of being unable to stop			0.564		
6	The early use of morphine makes it difficult to use any other treatment in severe pain			0.525		
	**“Other (non-dependence) risks” (eigenvalue = 1.111, % variance explained = 5.8)**					
7	IV administration is more effective than oral administration				0.593	
20	Risk of respiratory depression				0.613	
24	Risk of urinary retention				0.668	
	**“External (non-operational) reasons” (eigenvalue = 1.060, % variance explained = 5.6)**					
8	The patients are against the prescription of morphine					0.707
16	Sensation of pain decreases with age in the elderly, which does not justify its use					-0.501

Item numbering corresponds to the 24-item instrument. Results expressed as the highest factor loading for each component. Statistical analysis by factor analysis with varimax rotation and Kaiser normalization.

### Test-retest reliability

The convenience sample of thirty subjects was aged 44.5 ± 6.7 years (mean ± SD) and included 26 nurses and 4 doctors. Offering the same condition to complete the shorten questionnaire, a strong Pearson correlation was found between the test and the retest sample (*r* = 0.72, p < 0.001). The shortened instrument showed an ICC of 0.797 (95% CI, 0.630 to 0.911, P < 0.001) and a Cronbach’s α of 0.797, indicating that the internal consistency remained stable after removing 5 items.

## Discussion

To our knowledge, the AUM is the first instrument to evaluate the attitudes and beliefs of French-speaking health professionals regarding the prescription and administration of morphine. Overall, our results suggest that the shortened 19-item version of the AUM is a valid and reliable instrument that can be easily and quickly answered by health professionals without the need for specific filling instructions.

For over twenty years, several studies have tried to assess the attitudes of health professionals regarding morphine administration for pain management among hospitalized patients. The instrument of Brydon & Asbury [18] assesses the beliefs and attitudes of health professionals to relieve pain among adult surgical patients but, to our knowledge, this instrument has never been psychometrically evaluated. The instrument of Broekmans et al. [5] is based on the one of Brydon and Asbury and assesses the nurses’ attitudes towards pain relief using morphine. It consists of nine questions on addiction, side effects and use of opioids with the answers on a Likert scale ranging from 1 = “Totally agree” to 5 = “Totally disagree”. The authors reported a Cronbach’s α of 0.70, but again little psychometric data was provided. Finally, Edwards and al. assessed the determinants of registered nurses’ intention to administer opioids to patients presenting with pain [6] using an instrument (Pain Management Survey) consisting of 28 items and a 5-point Likert scale for response options ranging from 1 = “Totally disagree” to 5=”Totally agree”. The authors reported a Cronbach α of 0.78 but again no further psychometric information was provided. Indeed, according to several authors [4,12], further research is needed to assess attitudes towards the prescription and administration of morphine and its derivatives by health professionals.

### Original 24-item instrument

The 24-item instrument showed good practicality, with no floor or ceiling effect; conversely, almost one quarter of participants had at least one missing answer, thus precluding the calculation of the overall score. This value is close to the ones reported for other instruments [31] and suggests that the longer the instrument, the more likely the participants will miss one answer.

Reliability and internal consistency were also adequate, as indicated by an ICC of 0.69 and a Cronbach’s α of 0.700. Still, some items showed a poor correlation with the total and their deletion led to an improvement in Cronbach’s α. Factor analysis also showed that two items had communalities below the threshold value of 0.35. Overall, the analysis suggested that the 24-item instrument could be shortened without losing its properties.

### Shortened 19-item instrument

The shortened 19-item instrument showed a slightly lower percentage (19.9%) of participants with at least one missing value. It can thus be reasonably expected that the shortened instrument will be easier and possibly also quicker to complete.

The shortened 19-item version of the AUM presented an intra-class correlation of 0.740 and a Cronbach’s α of 0.741, both of which can be considered as “substantial” [27,28]. The internal consistency of the shortened instrument (Cronbach’s α of 0.741) was higher than the instrument of Broekmans et al. (0.700) [5] but slightly lower than the instrument used by Edwards et al. (0.78) [6]. Still, considering the internal consistency obtained in the retest procedure (Cronbach’s α of 0.797), it can be reasonably inferred that the shortened version of the AUM performs as well as other instruments previously used to assess attitudes towards the prescription and administration of morphine and its derivatives by health professionals. Further, the shortened instrument might be easier to use, as it only consists of 19 items, versus 28 for the instrument used by Edwards and col. [6]. Finally, the shortened instrument also presented a satisfactory degree of conformity and of internal consistency, with a between-item variance of 1.611 [29].

Factor analysis showed the shortened version to perform slightly better than the original one (54.3% vs. 52.4% of the variance explained, using 5 components instead of 6). This result confirms that the instrument has adequate validity regarding the concepts of morphine use and administration and the perception of risks. The 19 items were grouped into five constructs, tentatively termed “risk of addiction/dependence”; “operational reasons for not using morphine”; “risk of escalation”; “other (non-dependence) risks” and “external (non-operational) reasons”. The first one, which also showed the highest eigenvalue, clustered all items pertaining to addiction/dependence risk, while a second one clustered items more related to somatic risk. These findings suggest that French-speaking health professionals consider separately the different risks related to morphine prescription, and that they appear to be more sensitive towards the risk of addiction than to somatic risk.

Similarly, while a single 18-item construct “morphine use and administration” was initially found [12], three different constructs were obtained. The first one, “risk of escalation” clustered items related to the fact that once a morphine treatment is initiated, a dose escalation is necessary with potential deleterious effects on life expectancy. The second construct, “operational reasons for not using morphine” clustered items related to perceived in-hospital obstacles to use morphine, such as its difficulty to dose and its association with end of life and palliative care. Finally, the third one “external (non-operational) reasons for not using morphine” clustered items such as the fact that patients are against the prescription of morphine.

Overall, our results suggest that the shortened version of the AUM might provide interesting information regarding beliefs, reasons for not using and perceived risks of using morphine among health professionals. The reliability and internal consistency is comparable for nurses and doctors, suggesting that it can be applied to both health professions. Its shorter size will also increase practicality, with a decreased time for completion and a lower rate of missing answers. Nevertheless, other hypotheses must be verified and further studies on the validity of the instrument must be conducted.

### Strengths and limitations

This study has some limitations worth mentioning. First, the participation rate (53.5%) was rather low, and it is possible that non-responders might have a different response pattern than responders. Still, this participation rate is comparable or even higher than the ones reported by other psychometric studies [32,33]. Second, the study was geographically limited, and the sample might not be representative of all Swiss French-speaking health professionals. Still, making such a psychometric study in a multicenter setting was beyond our logistic capacities, and it would be of interest to apply this instrument in other French-speaking regions. Third, it is possible that cultural dimensions might influence the psychometric value of the instrument. Again, only the application of the instrument in other settings will allow a precise evaluation of the cultural dimensions regarding the answers. Fourth, no information was collected regarding highest level of education. Still, as in order to work as a nurse/doctor all foreign diplomas have to be validated, it is likely that all participants had at least an educational level comparable to those of Swiss doctors and nurses. Fifth, the content and face validity have not yet been reviewed. It is currently being done by two nursing professors (experts in pain management) of the Applied University of Nursing sciences La Source in Lausanne. Finally, longitudinal studies assessing the results of the instrument before and after an educational intervention on the prescription and administration of morphine should also be considered [34].

Among the strengths, the study was conducted in a large sample (588), considerably higher than the recommended 5 participants per item [27] and also higher than previous studies [5]. Further, the test-retest analysis showed a Cronbach’s α of 0.797, higher than the values reported for other instruments [5,6].

## Conclusions

Overall, our results suggest that the shortened version of the French questionnaire on attitudes towards the use of morphine is a valid instrument to assess attitudes and beliefs towards the prescription and administration of morphine among health professionals. The instrument allows the assessment of the main attitudes that might deter health professionals from using morphine in pain management and can be used in different settings among French-speaking countries to compare attitudes and to assess the effectiveness of training and educational programs regarding the prescription and administration of morphine.

## Competing interests

The authors declare that they have no competing interests.

## Authors’ contributions

MF, HV and CB were responsible for the study conception and design. MF, CB and HV performed the data collection. MF and PMV performed the data analysis. MF and PMV were responsible for the drafting of the manuscript. HV, CB and MMSV made critical revisions to the paper for important intellectual content. All authors have read and approved this version of the manuscript.

## Pre-publication history

The pre-publication history for this paper can be accessed here:

http://www.biomedcentral.com/1472-6955/13/1/prepub

## Supplementary Material

Additional file 1 French version of the questionnaire Attitudes towards morphine use. Click here for file

Additional file 2 Shortened 19-item French version of the Attitudes towards morphine use questionnaire: internal consistency, stratified by profession (nurses and doctors). Click here for file
