# Supplementary material for: Psychometric evaluation of the French version of the questionnaire attitudes towards morphine use; a cross-sectional study in Valais, Switzerland
Source: BMC Nurs. 2014 Jan 10;13:1. doi: 10.1186/1472-6955-13-1 (PMC4029768; doi:10.1186/1472-6955-13-1)
Supplement: Additional file 1 — French version of the questionnaire Attitudes towards morphine use. [file 1472-6955-13-1-S1.doc]

**Additional file 1 :** French version of the questionnaire Attitudes towards morphine use.

Quelle est votre opinion au sujet de l’utilisation de la morphine comme analgésique et ses effets probables sur les personnes malades ?

|  | **Totalement en désaccord** | **Partiellement en désaccord** | **Ni en accord ni en désaccord** | **Partiellement d’accord** | **Totalement d’accord** |
| --- | --- | --- | --- | --- | --- |
| **A. LES EXPRESSIONS** |  | | | | |
| 1 Cela signifie que c’est grave | ⬜ | ⬜ | ⬜ | ⬜ | ⬜ |
| 2 Diminue le temps de vie | ⬜ | ⬜ | ⬜ | ⬜ | ⬜ |
| 3 Peut s’habituer rapidement et prend le risque d’augmenter la dose | ⬜ | ⬜ | ⬜ | ⬜ | ⬜ |
| 4 Une fois le traitement entrepris, il existe le risque de ne plus pouvoir l’arrêter | ⬜ | ⬜ | ⬜ | ⬜ | ⬜ |
| 5 Toutes les personnes peuvent prendre de la morphine indépendamment du type de douleur | ⬜ | ⬜ | ⬜ | ⬜ | ⬜ |
| 6 L’utilisation précoce de la morphine rend difficile le recourt à tout autre traitement en cas de douleur intense | ⬜ | ⬜ | ⬜ | ⬜ | ⬜ |
| 7 La voie veineuse est plus efficace que la voie orale | ⬜ | ⬜ | ⬜ | ⬜ | ⬜ |
| 8 Les malades sont contre la prescription de la morphine | ⬜ | ⬜ | ⬜ | ⬜ | ⬜ |
| 9 La prescription de la morphine signifie qu’il n’y a plus aucune espérance de vie | ⬜ | ⬜ | ⬜ | ⬜ | ⬜ |
| 10 Il existe d’autres médicaments plus efficaces, ce qui ne justifie pas son utilisation | ⬜ | ⬜ | ⬜ | ⬜ | ⬜ |
| 11 Il est difficile d’utiliser et de doser la morphine | ⬜ | ⬜ | ⬜ | ⬜ | ⬜ |
| 12 Pour certains types de douleurs il est nécessaire d’utiliser la morphine | ⬜ | ⬜ | ⬜ | ⬜ | ⬜ |
| 13 La morphine est un médicament de dernier recours | ⬜ | ⬜ | ⬜ | ⬜ | ⬜ |
| 14 On peut arrêter la prise de la morphine lorsqu’on en a envie | ⬜ | ⬜ | ⬜ | ⬜ | ⬜ |
| 15 La prescription de morphine doit être évitée pour les malades en phase terminale | ⬜ | ⬜ | ⬜ | ⬜ | ⬜ |
| 16 Pour les personnes âgées, la sensation de douleur diminue avec l’âge, ce qui ne justifie pas son utilisation | ⬜ | ⬜ | ⬜ | ⬜ | ⬜ |

|  | **Totalement en désaccord** | **Partiellement en désaccord** | **Ni en accord ni en désaccord** | **Partiellement d’accord** | **Totalement d’accord** |
| --- | --- | --- | --- | --- | --- |
| **B. LES RISQUES** |  |  |  |  |  |
| 17 Risque de toxicodépendance | ⬜ | ⬜ | ⬜ | ⬜ | ⬜ |
| 18 Risque de délire ou d’euphorie | ⬜ | ⬜ | ⬜ | ⬜ | ⬜ |
| 19 Risque de somnolence et sédation | ⬜ | ⬜ | ⬜ | ⬜ | ⬜ |
| 20 Risque de dépression respiratoire | ⬜ | ⬜ | ⬜ | ⬜ | ⬜ |
| 21 Risque sur le plan légal par rapport aux autres médicaments | ⬜ | ⬜ | ⬜ | ⬜ | ⬜ |
| 22 Risque de dépendance physique / psychologique | ⬜ | ⬜ | ⬜ | ⬜ | ⬜ |
| 23 Risque de discrimination | ⬜ | ⬜ | ⬜ | ⬜ | ⬜ |
| 24 Risque de rétention urinaire | ⬜ | ⬜ | ⬜ | ⬜ | ⬜ |

The items excluded after psychometric evaluation are in grey background.
