# Supplementary material for: Psychometric evaluation of the French version of the questionnaire attitudes towards morphine use; a cross-sectional study in Valais, Switzerland
Source: BMC Nurs. 2014 Jan 10;13:1. doi: 10.1186/1472-6955-13-1 (PMC4029768; doi:10.1186/1472-6955-13-1)
Supplement: Additional file 2 — Shortened 19-item French version of the Attitudes towards morphine use questionnaire: internal consistency, stratified by profession (nurses and doctors). [file 1472-6955-13-1-S2.doc]

**Additional file 2:** Shortened 19-item French version of the Attitudes towards morphine use questionnaire: internal consistency, stratified by profession (nurses and doctors)

|  |  | **Nurses** | **Doctors** |
| --- | --- | --- | --- |
|  |  | **Alpha if item deleted** | **Alpha if item deleted** |
| 1 | It means it is serious | 0.730 | 0.720 |
| 2 | It decreases life expectancy | 0.736 | 0.704 |
| 3 | [The patient] can get used quickly and one takes the risk of increasing the dose | 0.713 | 0.691 |
| 4 | Once treatment is initiated, there is the risk of being unable to stop | 0.721 | 0.707 |
| 6 | The early use of morphine makes it difficult to use any other treatment in severe pain | 0.721 | 0.708 |
| 7 | IV administration is more effective than oral administration | 0.739 | 0.727 |
| 8 | The patients are against the prescription of morphine | 0.738 | 0.738 |
| 9 | The prescription of morphine means that there is no life expectation | 0.737 | 0.715 |
| 11 | It is difficult to use and dose morphine | 0.728 | 0.719 |
| 13 | Morphine is a drug of last resort | 0.722 | 0.722 |
| 16 | Sensation of pain decreases with age in the elderly, which does not justify its use | 0.743 | 0.733 |
| 17 | Risk of drug addiction | 0.722 | 0.725 |
| 18 | Risk of delirium or euphoria | 0.729 | 0.752 |
| 19 | Risk of drowsiness and sedation | 0.730 | 0.757 |
| 20 | Risk of respiratory depression | 0.727 | 0.731 |
| 21 | Legal risk compared to other drugs | 0.724 | 0.719 |
| 22 | Risk of physical and/or psychological dependence | 0.720 | 0.727 |
| 23 | Risk of discrimination | 0.740 | 0.729 |
| 24 | Risk of urinary retention | 0.736 | 0.754 |

Item numbering corresponds to the original 24-item instrument. The English version of the statements has not been psychometrically validated; please refer to the annex for the valid French terms.
